# Longitudinal Changes in Sports-Related Concussions Across the COVID-19 Pandemic: An Interrupted Time Series Analysis

**DOI:** 10.51894/001c.164051

**Published:** 2026-07-31

**Authors:** Gennyka Liebenthal, Angela S. Lee, Jacek Cholewicki, Nathan Fitton, Matthew R. Saffarian, John M. Popovich

**Affiliations:** 1 College of Osteopathic Medicine, Michigan State University

## 27

### Background

Concussion epidemiology has been well described in athletic and clinical populations. However, the COVID-19 pandemic disrupted organized sports, recreational activities, and access to medical care, making it unclear how concussion occurrence evolved across the pandemic and subsequent recovery period. Examining sports-related concussions (SRCs) may provide insight into altered injury patterns.

### Objective

To examine longitudinal changes in monthly concussions and the proportion of SRCs, before and after the COVID-19 pandemic.

### Methods

Concussion-related encounters were identified via UM Health-Sparrow’s electronic medical records between January 2018-July 2023 using ICD codes. All encounters were verified via chart review. SRCs were defined as concussions sustained during organized/recreational sports or exercise participation. Interrupted time series analysis (ITSA) evaluated changes in concussion occurrence and the proportion of SRCs with March 2020 defined as the COVID-19 disruption. Encounters were limited to March 2018-February 2022 to account for possible seasonal influences. Statistical significance was set at p<0.05.

### Results

Of 6,628 queried potential encounters, 5,187 were confirmed as unique concussion encounters. 1,386 were outside the ITSA timeframe, leaving 3,801 encounters with 964 (25.4%) SRCs. Pre-pandemic, the mean monthly concussion count was 94.8±24.6, with 27.1%±8.1% SRCs, whereas post-pandemic, the mean was 63.6±21.2, with 19.4%±10.2% SRCs. There was a non-significant increasing pre-pandemic trend of 1.2 concussions/month (95%CI=[−0.1, 2.6], *p* =0.07). At the pandemic onset in March 2020, concussion encounters decreased significantly by 70.1 cases (95%CI=[−95.0, −45.3], *p* < 0.001), followed by a significant increase of 2.0 concussions/month (95%CI=[0.9, 3.2], p< 0.001). SRC trend didn’t increase during pre-pandemic (95%CI=[−0.50, 0.5], *p* = 0.92), but showed a significant decrease (-13.8%, 95%CI=[−22.3, −5.3], *p* = 0.001) at the onset. Although SRCs increased post-pandemic, the proportion did not return to pre-pandemic levels by February 2022.

### Discussion

Concussion occurrences were significantly affected during the pandemic, reflecting widespread disruptions to organized sports/recreational activities, and access to medical care. Post-pandemic trends suggest persistent changes rather than a uniform return to pre-pandemic levels, which may stem from changes in behaviors related to sports participation and healthcare utilization. These findings highlight the importance of continued surveillance in understanding concussion epidemiology and may inform injury prevention strategies as activities continue to normalize.

